# 

**Published:** 1880-01

**Authors:** 


Vol. 1.] TWO DOLLARS A YEAR. SINGLE COPIES 50 CENTS. [No. 1.

### THE ARCHIVES

OF

### COMPARATIVE MEDICINE AND SURGERY.

A Quarterly Journal

OF THE

ANATOMY, PATHOLOGY, AND THERAPEUTICS
OF THE LOWER ANIMALS.

*EDITED BY*

EDWARD C. SPITZKA, M. D.,

PROFESSOR OF COMPARATIVE ANATOMY AND EMBRYOLOGY, COLUMBIA VETERINARY COLLEGE.

**JANUARY, 1880.**

NEW YORK:

W. L. HYDE & CO., PRINTERS, NO. 22 UNION SQUARE.

1880.

